# Brain Correlates of the Alcohol Use Disorder Pharmacotherapy Response: A Systematic Review of Neuroimaging Studies

**DOI:** 10.3390/brainsci12030386

**Published:** 2022-03-14

**Authors:** Luiza Florence, Dângela Layne Silva Lassi, Guilherme T. Kortas, Danielle R. Lima, Cintia de Azevedo-Marques Périco, Arthur G. Andrade, Julio Torales, Antonio Ventriglio, Domenico De Berardis, João P. De Aquino, João M. Castaldelli-Maia

**Affiliations:** 1 Department of Neuroscience, FMABC University Center, Santo André 09060-870, SP, Brazil; luiza.florence@yahoo.com (L.F.); cazevedomarques@hotmail.com (C.d.A.-M.P.); aandrade@usp.br (A.G.A.); 2 Department of Psychiatry, Medical School, University of São Paulo, São Paulo 05508-060, SP, Brazil; dangela.lassi@gmail.com (D.L.S.L.); gtkortas@gmail.com (G.T.K.); elleruiz@hotmail.com (D.R.L.); 3 Department of Psychiatry, National University of Asunción, San Lorenzo 2064, Paraguay; juliotorales@gmail.com; 4 Department of Clinical and Experimental Medicine, University of Foggia, 71122 Foggia, Italy; a.ventriglio@libero.it; 5 Mental Health Center of Giulianova, Asl Teramo, 64021 Giulianova, Italy; domenico.deberardis@aslteramo.it; 6 Department of Neurosciences and Imaging, University “G. D’Annunzio” Chieti, 66100 Chieti, Italy; 7 International Centre for Education and Research in Neuropsychiatry, University of Samara, 443100 Samara, Russia; 8 Department of Psychiatry, Yale University School of Medicine, New Haven, CT 06510, USA; joao.deaquino@yale.edu

**Keywords:** alcohol, pharmacotherapy, neuroimaging, naltrexone, acamprosate, disulfiram, gabapentin

## Abstract

Background: Although Alcohol Use Disorder (AUD) is highly prevalent worldwide, treating this condition remains challenging. Further, potential treatments for AUD do not fully address alcohol-induced neuroadaptive changes. Understanding the effects of pharmacotherapies for AUD on the human brain may lead to tailored, more effective treatments, and improved individual clinical outcomes. Objectives: We systematically reviewed the literature for studies investigating pharmacotherapies for AUD that included neuroimaging-based treatment outcomes. We searched the PubMed, Scielo, and PsycINFO databases up to January 2021. Study eligibility criteria, participants, and interventions: Eligible studies included those investigating pharmacotherapies for AUD and employing functional magnetic resonance imaging (fMRI), positron emission tomography (PET), single-photon emission computed tomography (SPECT), and/or proton magnetic resonance spectroscopy (H-MRS). Study appraisal and synthesis methods: Two independent reviewers screened studies’ titles and abstracts for inclusion. Data extraction forms were shared among all the authors to standardize data collection. We gathered information on the following variables: sample size; mean age; sociodemographic and clinical characteristics; alcohol use status; study design and methodology; main neuroimaging findings and brain-regions of interest (i.e., brain areas activated by alcohol use and possible pharmacological interactions); and limitations of each study. Results: Out of 177 studies selected, 20 studies provided relevant data for the research topic. Findings indicate that: (1) Acamprosate and gabapentin may selectively modulate limbic regions and the anterior cingulate cortex; (2) Naltrexone and disulfiram effects may involve prefrontal, premotor, and cerebellar regions; (3) Pharmacotherapies acting on glutamate and GABA neurotransmission involve primarily areas underpinning reward and negative affective states, and; (4) Pharmacotherapies acting on opioid and dopamine systems may affect areas responsible for the cognitive and motor factors of AUD. Limitations: Most of the studies were focused on naltrexone. A small number of studies investigated the action of disulfiram and gabapentin, and no neuroimaging studies investigated topiramate. In addition, the time between medication and neuroimaging scans varied widely across studies. Conclusions: We identified key-brain regions modulated by treatments available for AUD. Some of the regions modulated by naltrexone are not specific to the brain reward system, such as the parahippocampal gyrus (temporal lobe), parietal and occipital lobes. Other treatments also modulate not specific regions of the reward system, but play a role in the addictive behaviors, including the insula and dorsolateral prefrontal cortex. The role of these brain regions in mediating the AUD pharmacotherapy response warrants investigation in future research studies.

## 1. Introduction

Alcohol use disorder (AUD) is a chronic, relapsing-remitting disorder that is strongly associated with both medical and psychiatric conditions and affects over 107,460,000 persons—1.4% of the population—worldwide [1]. The hallmark of AUD is an inability to control alcohol use despite its negative consequences [1,2,3]. A wealth of evidence indicates that neurobiological abnormalities play an essential role in the development and maintenance of AUD, as well as in the recovery from this condition [4,5]. From a neurobiological perspective, AUD involves counter-adaptations to chronic alcohol exposure, with broad alterations in the neurotransmission of gamma amino butyric acid (GABA), glutamate, dopamine, serotonin, and opioid systems. Thus far, only three drugs have been approved by the Food and Drug Administration (FDA) to treat AUD: naltrexone (NTX), approved in 1994; acamprosate (ACA) approved in 2004; and disulfiram (DSF), approved in 1951. Although there are many treatments for AUD in the drug development pipeline, the currently available medications have shown modest efficacy for promoting alcohol abstinence [6].

Neuroimaging techniques may be useful in assessing the changes in specific brain areas related to AUD, such as reward and motivation systems, executive functions, and inhibitory control. Further, neuroimaging techniques can be used to explore the impact of pharmacotherapies on alcohol-induced neuroadaptations (e.g., activation of limbic areas) [4,5]. These neuroimaging techniques include Functional Magnetic Resonance Imaging (fMRI), which measures regional blood flow as a proxy of neuronal activity; localized Magnetic Resonance Spectroscopy (MRS), which measures regional intrinsic brain metabolite levels; Positron emission tomography (PET), which uses radionuclides to assess changes in receptor availability and neurotransmitter release; and Single Photon Emission Computed Tomography (SPECT), which employs gamma rays to detect changes in cerebral blood flow, thereby yielding quantitative information on selected molecules within defined brain regions [7,8,9,10].

This systematic review summarizes evidence from pharmacotherapeutic studies on AUD that have employed neuroimaging-based biomarkers. First, we review mechanisms of action of treatments for AUD, including their interactions with various neurotransmitter systems. Second, we appraise the evidence on neuroimaging biomarkers of the AUD treatment response. Finally, we provide conceptual and methodological insights to promote the development AUD pharmacotherapies, considering potential predictors of treatment responses and other individual-level factors.

### Pharmacotherapies for Alcohol Use Disorder

To this date, FDA has approved three treatments for AUD: Naltrexone (NTX), acamprosate (ACA), and disulfiram (DSF) [11,12,13,14,15]. Notably, the American Psychiatric Association (APA) Practice Guideline for the Pharmacological Treatment of Patients with Alcohol Use Disorder, released in 2018, recommended the off-label use of topiramate and gabapentin (GBP) [11]. Collectively, these drugs are the most widely used clinically, and here we synthesize data from neuroimaging studies that have administered NTX, ACA, DSF, gabapentin, or topiramate.

NTX is an opioid antagonist that acts on µ, δ, and κ receptors and was originally developed to treat opioid use disorder. Although a remarkable number of studies have investigated NTX for the treatment of AUD in the last decades, NTX was approved by the FDA in 1994 for the treatment of AUD. By blocking µ, δ, and κ receptors, NTX influences dopamine levels in the mesolimbic pathway, reducing the hedonic effects of alcohol, and curbing heavy drinking. However, a substantial proportion of patients with AUD do not respond adequately to NTX [12], with emerging evidence suggesting that adequate responses may be contingent on pharmacogenetic mechanisms [13,14]. 

ACA, tested in clinical trials since the 60s, was first commercialized in France in 1989, and became the third FDA-approved pharmacotherapy for AUD in 2004 [15]. It is a synthetic compound with a chemical structure akin to that of the endogenous amino acid homotaurine, which is a structural analogue of GABA and taurine. ACA boosts GABA activity while decreasing glutamate activity in the Central Nervous System (CNS), leading to a reduction of the activity of N-methyl D-aspartate (NMDA) receptors. ACA may also exert its effects partly by binding to CNS calcium channels. Collectively, the GABA and glutamate activity implicated in AUD are modulated by ACA [16,17,18,19,20]. 

Although DSF was the first drug approved by the FDA [12], it is currently considered a third line treatment for AUD [11]. DSF acts mainly by inhibiting acetaldehyde dehydrogenase (ALDH) and impeding the conversion of acetaldehyde to acetate, thereby causing acetaldehyde accumulation, causing aversive effects—such as nausea, vomiting, headache, vasodilation, hypotension, tachycardia, and confusion [12]. The effects of DSF can last for up to 2 weeks after the interruption of the medication, so any alcohol intake is avoided during this period [21]. Despite its protracted effects, the evidence supporting the abstinence-promoting effects of DSF is modest. However, DSF has been associated with a significant reduction of drinking days [22]. 

GBP and topiramate are also commonly used to treat AUD as off-label treatments [11]. Like GBP, topiramate acts on the GABAergic and glutamatergic systems of the CNS. The main mechanism of action of topiramate is the inhibition of dopamine release in the mesocorticolimbic system [12,23]. Converging evidence shows that these drugs attenuate alcohol withdrawal and may prevent relapse [24,25]. Additionally, the anti-craving effects of topiramate have been associated with withdrawal suppression, abstinence promotion, and fewer drinks on drinking days [12]. GBP is believed to act by blocking a specific α-2d subunit of voltage-gated calcium channels at selective presynaptic sites and, as a result, to modulate GABA neurotransmission indirectly. Besides reducing alcohol use, GBP has also been shown to promote a significant improvement in cognitive functioning, insomnia, and compulsive behaviors among persons with AUD [23].

## 2. Methods

### 2.1. Eligibility

This review was conducted according to the Preferred Report Items for Systematic Reviews and Meta-analysis (PRISMA). The corresponding checklist is available in Supplementary File 1. We included original studies, published in English, reporting on pharmacotherapies of AUD, and using the following neuroimaging techniques: fMRI—with or without blood oxygen level-dependent (BOLD)—and/or PET and/or SPECT.

We excluded case reports; review articles; commentaries; studies in languages other than English; animal studies; post-mortem studies; studies including only healthy subjects; studies with drugs not approved by the FDA or not included in the Guideline for the Pharmacological Treatment of Patients with Alcohol Use Disorder (APA, 2018), thereby keeping only drugs commonly used for AUD treatment.

### 2.2. Search Strategy

We searched the PubMed, Scielo, and PsycINFO databases and reviewed findings of search-input up to 29 January 2021, using the following terms: (naltrexone OR disulfiram OR topiramate OR acamprosate OR gabapentin) and (alcoho*) and (neuroimage OR neuroimaging OR magnetic resonance OR SPECT OR fMRI OR functional magnetic resonance OR pet OR positron emission tomography). 

### 2.3. Study Selection and Data Extraction

Two independent reviewers (LF and JMCM) screened study titles and abstracts for inclusion with a consensus on selection criteria. Data extraction forms were developed and circulated to the author group before piloting and refining. All data were extracted by one of the reviewers (LF) and checked by a second reviewer (JMCM). The same reviewers resolved any remaining inconsistencies. 

We gathered information on the following variables: sample size and mean age; main sociodemographic and clinical characteristics; alcohol use status; study design and methodology; main neuroimaging findings and brain-regions of interest (i.e., brain areas activated by alcohol use and possible pharmacological interactions); study challenges and limitations. 

### 2.4. Registration

The methodology of this systematic review was registered in the Open Science Framework (OSF), under the following code: e67qp (2 August 2020). Available online: https://osf.io/e67qp/ (accessed on 2 November 2021).

## 3. Results

**Figure 1:**
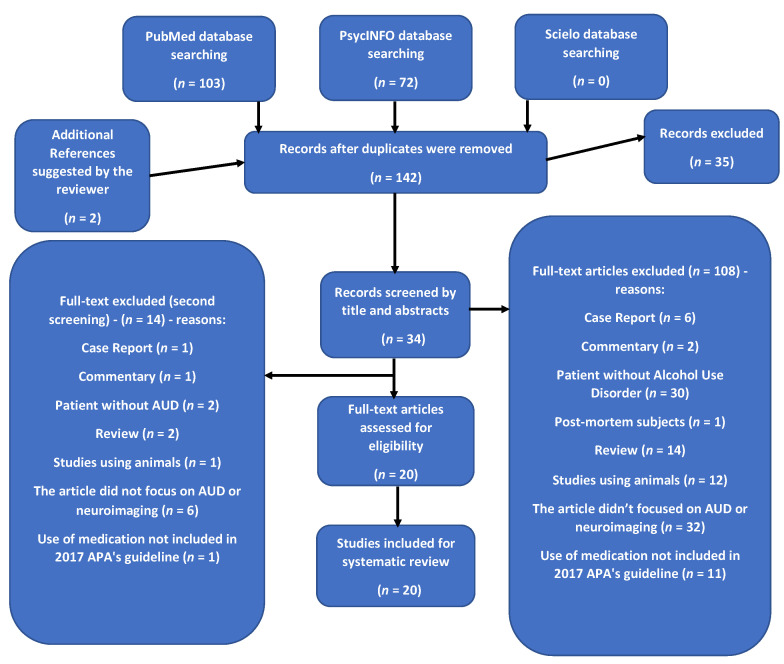
PRISMA illustrating the screening process applied in the study.

After removing duplicates, we identified 140 records, and 18 neuroimaging studies were included in the final review (see Figure 1 for the PRISMA diagram). The selected 18 studies included *n* = 918 participants (Table 1). Among these studies, 13 used fMRI, two studies used PET, one study used SPECT, and two studies used H-RMS (Table 2). The pharmacotherapies investigated were: NTX (14) [11,12,26,27,28,29,30,31,32,33,34,35,36]; ACA (4) [16,27,37,38]; DSF (1) [39]; and GBP (1) [40]. None of the studies included topiramate. Finally, one single study compared the effects of NTX to ACA [27]. Pharmacotherapies employed in each study are shown in Table 1. 

### 3.1. Sample Characteristics 

The mean age of participants was 40 years old. Only four studies included persons younger than 35 years old: Schacht et al. [14]; Lim et al. [35]; Myrick et al. [28]; and Umhau et al. [38]. As expected, participants were more likely to be men in 17 out of 18 studies [13,14,26,27,28,29,30,31,32,33,34,35,36,37,38,39,40]. Almost 53% of the individuals diagnosed with AUD were tobacco smokers [11,12,16,26,27,29,31,34,37,38,42]. Although education history was not reported in all studies, available data indicated that persons with AUD had a minimum of 10 years of formal education. The alcohol use status of the study participants was highly heterogeneous, ranging from persons who were actively drinking, to persons undergoing current or recent alcohol withdrawal, to persons experiencing sustained abstinence from alcohol [13,14,16,18,26,27,28,29,30,31,32,33,34,36,37,38,39,40,41]. The studies included treatment-seeking patients [13,26,27,29,31,33,34,37,38,40], non-treatment-seeking patients [14,28,35], and subjects in acute treatment (currently or recently) [30,32,36,39]. 

### 3.2. Main Findings

#### 3.2.1. Duration of Treatment and Study Design

Table 2 shows the characteristics of each study in terms of duration and type of treatment, the duration of follow-up, and the measures used to evaluate AUD treatment efficacy. In all the studies included, pharmacotherapies for AUD were administered for at least one week, with some studies providing treatment for up to 18 months [13,16,27,34,37,38,39,40]. The time elapsed between medication administration and the neuroimaging scan varied from a few hours to a few days [26,28,29,30,32,33,35,36]. 

Only eight studies had a longitudinal design. Longitudinal investigation in these studies included at least four encounters. Schacht et al. (2017) [13] reported a set of nine visits to evaluate ongoing treatments. Lukas et al. (2013) [32] performed weekly visits in the first month following the medication intake (4 visits). Bach et al. (2019) [34] reported a follow-up of three months after the two weeks of treatment. Spagnolo et al. (2014) [33] set a 3-week follow-up; Mann et al. (2014) [27] performed a 6-month counseling and one-year follow-up. Priscindaro et al. (2021) [41] conducted a 16-week randomized clinical trial. Finally, Gilman et al. (1996) [39]. Nestor et al. (2019) [36], Weerts et al. (2008) [29]. Schacht et al. (2013) [40], and Morris et al. did not specify the duration of their studies follow-up [31].

In most of these studies, the neuroimaging scans were conducted across two or more sessions (11 out of 18 studies) [11,27,29,30,31,32,34,35,36,37,38,41]. Five studies performed the scan at baseline and two weeks later [13,14,27,34,37]. None of the studies performed brain scans after 30 days from pharmacotherapy [14,16,26,28,33,39,40].

#### 3.2.2. Neuroimaging Findings of the Pharmacotherapy Response

Naltrexone was investigated in 14 studies. Approximately 30 brain regions were found to be deactivated by the administration of NTX in individuals with AUD [13,14,26,27,28,29,30,31,32,33,34,35,36]. The Ventral Striatum (VS) was a prominent area of interest for studies involving NTX. For instance, following the NTX administration, lower activity in the right vs. was associated with fewer days of heavy drinking [11]. One study also showed an interaction between this treatment and the A118G genotype on orbitofrontal cortex (OFC) activation and that the human dopamine transporter (DAT1) would moderate NTX effects on vs. and medial prefrontal cortex (mPFC) activation [14]. In a study comparing NTX vs. ACA, the authors observed that among areas activated by alcohol-related cues such as VS, as the cue reactivity increased, the risk of relapse decreased in patients assigned to NTX compared to those treated with ACA [27]. Another study showed that NTX with or without ondansetron also lowered alcohol cue-induced activation of the vs. [28]. 

In a more recent study, NTX seemed to enhance OFC activity. The AUD group exhibited greater activation in the anterior cingulate cortex (ACC) and inferior frontal gyrus during the treatment [36]. In addition, NTX increased functional connectivity between right vs. and OFC [35]. Studies focused on basal ganglia and the temporal lobe, reported significant regional cerebral brain-flow decreases in the basal ganglia (right and left) and the left mesial temporal cortex after the administration of NTX [30]. Another study investigated cue reactivity in a whole-brain analytic strategy, focusing on the putamen, and reported a reduction in relapse-risk of heavy drinking probably due to attenuation in left putamen cue reactivity, which also may be observed in the thalamus as related to an attenuation of drug reward and drug reactivity, as well as in the ventral pallidum [34]. NTX also normalized the right amygdala activation in the intervention group and modulated task-related activity in the mPFC [26]. 

Weerts et al. [29] found that binding at PET in the thalamus and cerebellum was lower than in other regions of interest (ROIs). Moreover, binding was only partially inhibited by NTX across all the regions. Lukas et al. [32] studied the extended-release NTX (XR-NTX) effects and, differently from NTX, it did not modulate key areas such as the amygdala; however, this study described: (a) a decrease of frontal lobe activation by XR-NTX, which is an area intimately related to different addictive behaviors involving the parietal lobe, occipital lobe, and limbic regions; (b) activation in the limbic and grey matter regions and the visual cortex with the presentation of alcohol-related cues. Other evidence [31] has shown that NTX reduced (or “normalized”) local efficiency in the medial OFC, supplementary motor area, left middle frontal gyrus, left middle occipital gyrus, left para-hippocampus; also, the olfactory bulb node showed an increased and decreased local efficiency among participants with AUD before and after NTX administration, respectively.

Three studies explored the ACA action in the brain employing fMRI [16,27,37], and one using proton magnetic resonance spectroscopy (H-MRS) [38]. A decrease in central glutamate levels in the cingulate cortex was observed by introducing ACA treatment at the onset of alcohol abstinence. In contrast, the level of glutamate in the cerebral spinal fluid remained unaffected in one study [38]. Frye et al. [16] reported a reduction of glutamate levels in the midline ACC and cerebral spinal fluid, similar to the level of healthy subjects investigated with ACA treatment. In addition, patients reported an alleviation of craving with reduced glutamate levels [16]. Comparing ACA with NTX, a reduced risk of relapse was found in patients with NTX compared to those assigned to ACA [27]. ACA treatment also had no additional effect on cue-related brain activity compared with conventional nonpharmacological intervention for AUD [37]. 

Only one study [39] analyzed the effects of DSF on the human brain. A PET scan was performed after 30 days of abstinence from alcohol and the study found decreased values in radioactive markers in global and specific regions (medial frontal, dorsolateral frontal, orbito-medial frontal, posterior superior temporal, inferior parietal, and cerebellar hemispheres) among patients receiving DSF, without statistical testing [39]. 

GBP was investigated in three studies [18,40,41]. Using MR spectroscopy in a longitudinal design, before treatment and 14 days following randomization in a 16-week trial, Priscindaro et al. (2021) found higher levels of GBP in the dorsal ACC [41]. In another 16-week trial, GBP was co-administered with flumazenil (FMZ) [40]: An fMRI scan was performed between the second and third week of the study using exposure to images of alcoholic and non-alcoholic beverages. Similarly, higher levels of GBP were observed in the dorsal ACC. Finally, in a 1-week trial, individuals who received GBP reported markedly lower levels of glutamate in the frontal white matter than those who had not [18].

## 4. Discussion

**Figure 2:**
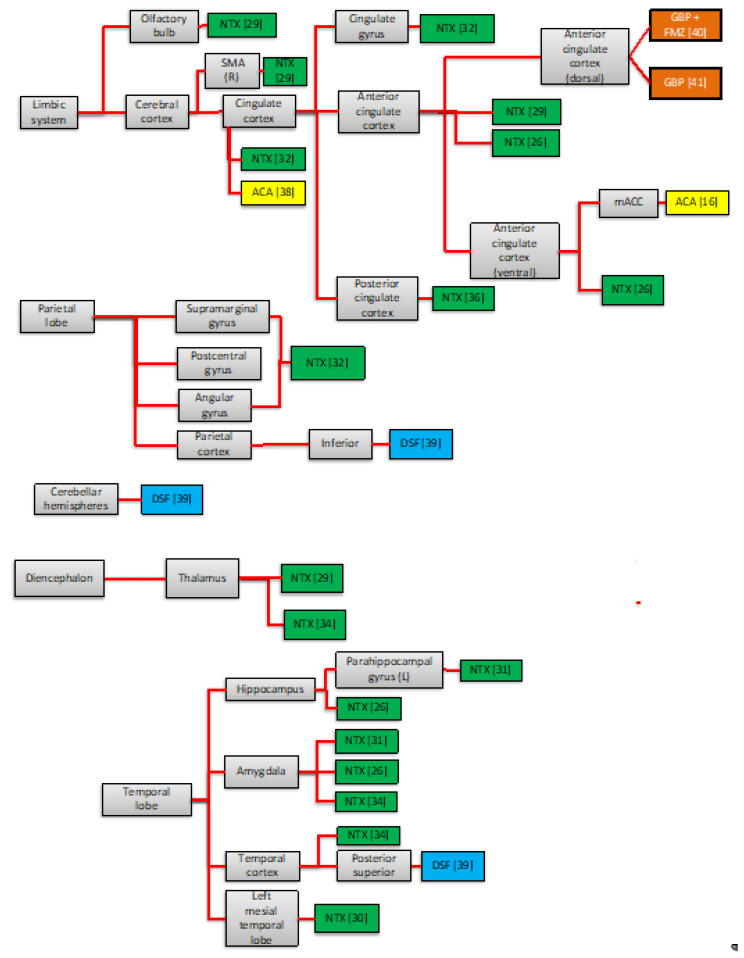

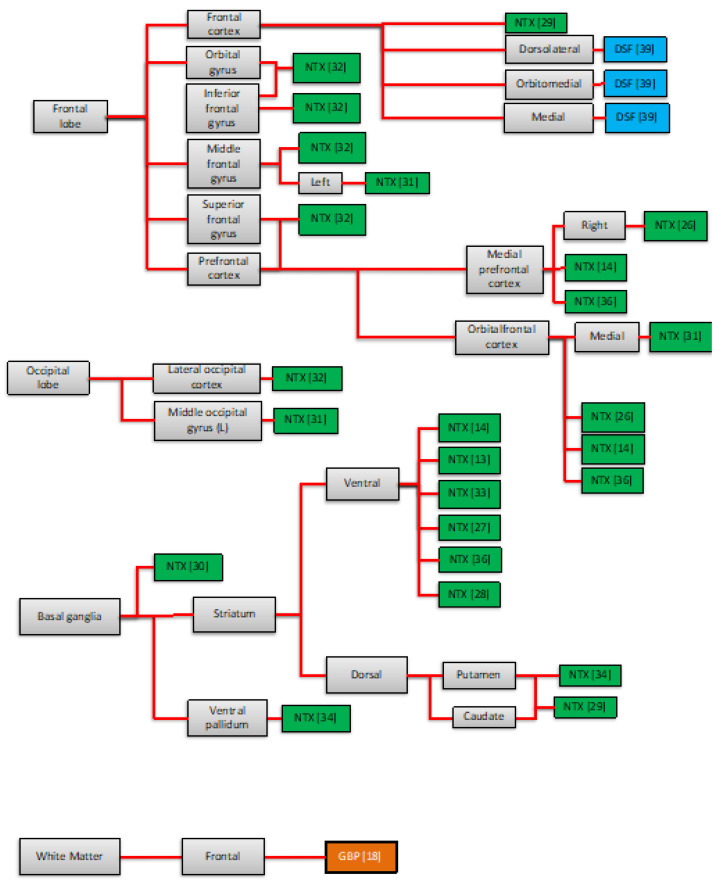
Neural areas affected by pharmacological treatment compared to placebo in neuroimaging studies. SMA = Supplementary Motor Area—NTX = Naltrexone—ACA = Acamprosate—DSF = Disulfiram—GBP = Gabapentin—FMZ = Flumazenil—ODT = Ondansetron; Mann et al. [27] and Langosch et al. [37]: No areas modulated by ACA; No Deactivation was correlated with NTX [13,14,26,27,28,29,30,31,32,34], ACA [16,38], DSF [39]. Activation was correlated with NTX [33,36] and GBP [40] use. Higher [41] and lower levels of glutamate were found with GBP use [18].

This review aimed to identify brain imaging correlates of the AUD pharmacotherapy response. Studies have shown that NTX, ACA, DSF, and GBP modulate either limbic or brain reward networks (e.g., limbic system, prefrontal cortex, amygdala, basal ganglia), which are implicated in the pathophysiology of AUD. Surprisingly, these drugs modulated other areas not traditionally associated with the pathophysiology of substance use disorders (e.g., parietal lobe, diencephalon, and occipital lobe). Mechanisms of action can range from actions in specific areas to coordinated actions across multiple areas (See Figure 2).

### 4.1. Areas Traditionally Involved in the Neurobiology of AUD

As expected, the limbic system was investigated by several studies reporting on medications for AUD, as included in this review. The limbic system contains complex networks involved in mood and behavioral regulation [43]. The activation or increased function of limbic areas could be observed with the presentation of alcohol-related cues (posterior and ACC [23,32]); during the motor impulse control in the AUD group [36]; and even in baseline evaluations among participants with AUD (supplementary motor area, olfactory bulb node [31], ACC, posterior cingulate cortex [27]). Conversely, the deactivation of these areas could be noted after the treatment with NTX [31,34,39], after XR-NTX treatment [29], and also after GABA + flumazenil treatment [21]. These findings are consistent with a superior alcohol treatment response since the dorsal ACC is related to alcohol cue-related brain activity and negative reinforcement [40]. Findings from the literature suggest that ACA and GBP may selectively modulate limbic regions and the ACC [18]. GBP is more associated with reducing cortical glutamate than elevating GABA in the frontal region [41]. 

It is well known that the temporal cortex is rich in opioid receptors. This area is related to the emotional memory and obsessive-compulsive behavior, impacting on craving symptoms [44]. Studies focusing on this area found significant regional cerebral brain flow decreases in the left mesial temporal cortex [30], in the right amygdala [26,29,31], and left para-hippocampus [26,31] after NTX administration. 

Some frontal lobe areas are linked to cognitive and motivational functions, which modulate drug reinforcement and processes to control and inhibit prepotent responses [45]. For example, NTX modulated task-related activity in the mPFC [26]. In addition, other evidence revealed an increased activation after drug cues, decreased frontal lobe activations after XR-NTX administration [32], and decreased local activation in the medial OFC among participants with AUD after NTX administration [31].

The ventral striatum (VS) in the basal ganglia has been shown to be a relevant area of interest of the NTX effect in the studies. It has been observed, for example, that lower activity in the right ventral after NTX administration was associated with fewer days of heavy drinking [46]. Furthermore, NTX response in the vs. was greater than in placebo groups, but not in the amygdala, leading to the conclusion that NTX modulates the vs. [30,33,34]. Moreover, it has been described that treatment with NTX deactivated vs. in three other studies [27,28,35].

### 4.2. Other Neural Areas

In an animal study [47], it was observed that the parietal lobe plays a critical role in remapping abstract valuation to concrete action. XR-NTX also deactivated the parietal lobe transmission in an included study [32].

Neuroimaging data have also underscored that the cerebellum is consistently activated when drug-associated cues are presented. Findings point out that this cue exposure would trigger a cerebellum-generated prediction of drug availability that would activate the preparation of the brain networks required to trigger drug-seeking and drug use behaviors [48]. In an included study using PET techniques, Cerebellum μ-OR biding potential was only partially inhibited by NTX [29].

The diencephalon is considered another area of interest. Patients with AUD presented higher activation in the thalamus [27]. An attenuation on cue reactivity was observed in the thalamus after administration of NTX, which might be related to NTX-related attenuation of drug reward and drug reactivity systems [29,34]. 

The occipital cortex is implicated in behavioral inhibition and motor impulse control [36]. This area was activated after visual alcohol cues in a trial, deactivated after a single oral NTX [31], and after two weeks of a single injection of XR-NTX. NTX also increased functional connectivity between the right vs. and OFC [35], which might mean greater activation of self-control networks in the brain following NTX treatment [35]. 

All these findings should encourage the extension of neuroimaging studies to those neuronal circuits not traditionally associated with AUD mechanisms. Given prior findings suggesting biological plausibility, the impact of AUD pharmacotherapies on these circuits warrants investigation.

### 4.3. Implications

Findings from this review show the importance of a baseline neuroimaging scan and a defined duration of medication treatment to allow a precise evaluation of the AUD pharmacotherapies. In addition, a longitudinal design with a defined follow-up period is suggested to examine clinical outcomes, brain changes, and their correlation with the ongoing treatments. Affective and cognitive outcomes should be measured to gather more relevant data since their brain correlates seem to be modulated by pharmacological treatments.

In conclusion, these insights may encourage future studies to examine the relationship between treatments, neuroimaging biomarkers, and specific affective, cognitive, and motor symptom clusters of AUD.

### 4.4. Limitations

Despite its notable strengths, this review has limitations that are largely related to the original studies included. Most of the studies were focused on NTX, few studies investigated DSF and GBP, and no neuroimaging studies investigated the treatment response to topiramate. Unfortunately, there was a significant variation regarding the time of neuroimage-scan after the exposure to treatments. The scarcity of studies with longer follow-up significantly limits inferences about the long-term effects of available drugs to treat AUD on the human brain: out of fifteen studies, only six reported follow-up assessments. Further studies investigating long-term neural and clinical effects of pharmacotherapies for AUD treatment are needed.

### 4.5. Future Directions

A few neuroimaging studies have investigated the role of the insula (limbic system) and dorsolateral prefrontal cortex in the treatment of AUD—warranting investigation in future studies. Notably, these areas are both activated in the presence of alcohol cues [49,50]. For instance, the dorsolateral prefrontal cortex is associated with selective attention, inhibition, and control processes, so its activation might predict a return to drinking [40,49,50]. Thus far, none of the studies available have investigated whether the available treatments (NTX, ACA, DSF, GBP) can (de-)activate the dorsolateral prefrontal cortex. Likewise, the insula and its extensions to temporal cortices have been implicated in the pathophysiology of AUD [51]. For example, a meta-analysis reported significant connectivity between the insula and the anterior dorsal striatum, an area responsible for habit formation [52]. Another study reported connections between the insula and other limbic structures, such as the ventromedial prefrontal cortex, amygdala, and VS, describing its involvement with craving [49,53]. Those areas may be important to further the addiction cycle due to their activation upon exposure to alcohol cues. Therefore, further studies are needed to clarify their significance to the neural circuitry of AUD, and the role of other less studied areas in the pathophysiology of addiction—such as the parietal lobe, cerebellum, diencephalon, and occipital lobe. 

## 5. Conclusions

We identified key brain regions modulated by commonly used pharmacotherapies for AUD. Notably, some of the regions modulated by NTX are not specific to the brain reward system, like the parahippocampal gyrus (temporal lobe), parietal and occipital lobes. Other treatments also modulate not specific regions of the reward system but play a role in the addictive behaviors, like the insula and DLPFC. Those areas warrant further investigation as biomarkers of the AUD treatment response in further longitudinal studies, such that they may help parse out responders from non-responders, ultimately improving treatment outcomes. 

## Figures and Tables

**Table 1:** Total sample in each study, by medication received and placebo.

Reference	NTX	XR-NTX	NTX + ODT	ODT	ACA	DSF	GBP	GBP + FMZ	PLA	IWT	HCS
Bach et al. (2019) [34]	22									13	35
Catafau et al. (1999) [30]	29										
Frye et al. (2016) [16]					9						16
Gilman et al. (1996) [39]						11					
Langosch et al. (2012) [37]					15				14		
Lim et al. (2019) * [35]	41								41		
Lukas et al. (2013) [32]		15							13		
Mann et al. (2014) [27]	36				28						
Meyerhoff et al. (2018) [18]							13				
Morris et al. (2017) [31]	45								48		
Myrick et al. (2008) [28]	23		20	23					24		17
Nestor et al. (2019) [36]	NA								NA		35
Prisciandaro et al. (2021) [41]							31		37		
Savulich et al. (2017) * [26]	18								18		21
Schacht et al. (2012) [14]	33								39		
Schacht et al. (2013) [40]								28	20		
Schacht et al. (2017) [13]	76								56		
Spagnolo et al. (2014) [33]	31								32		
Umhau et al. (2010) [38]					15				18		
Weerts et al. (2008) [29]	36										
TOTAL	390	15	20	23	67	11	44	28	360		124

Naltrexone, NTX; Extended-Release Naltrexone, XR-NTX; Ondansetron, ODT; Acamprosate, ACA; Disulfiram, DSF; Gabapentin, GBP; Flumazenil, FMZ; Placebo, PLA; NA = Not available information. Intensive Withdrawal Treatment, IWT; Healthy Control Subjects, HCS; * Individuals with AUD were administered a placebo or naltrexone in a counterbalanced order.

**Table 2:** Treatment characteristics of each study, including duration, neuroimage exam utilized, follow-up, and measures used.

Reference	Treatment	Neuroimage Study	Follow-Up	Alcohol Use Scales
Bach et al. (2019) [34]	NTX 21 days	fMRI at baseline (after 2–4 weeks of controlled abstinence) and after 2 weeks of treatment	3 month follow-up	BDI, ADS Score, OCDS Score, CIWA-Ar, TLFB
Catafau et al. (1999) [30]	NTX 1 day	SPECT on the tenth day of abstinence and on day 12 after 150 mg NTX (oral)	NA	MAIPY, MTAA
Frye et al. (2016) [16]	ACA 4 weeks	H-MRS shortly after admission and after 4 weeks of NTX treatment	NA	BDI-II, PHQ-9, TLFB, DSLD, PACS, AUQ, CIWA
Gilman et al. (1996) [39]	DSF 30 days	PET Scan was conducted after at least 30 days of sobriety, except for one patient	NA	LTAC, YHD
Langosch et al. (2012) [37]	ACA 2 weeks	fMRI before treatment initiation and after 2 weeks of treatment	NA	PSS, BDI-II, CIWA
Lim et al. (2019) [35]	NTX 8 days	One fMRI session after 4 days of NTX and another after 4 days on placebo	NA	AUDIT, TLFB, DD, DPDD
Lukas et al. (2013) [32]	XR-NTX 4 weeks	fMRI immediatly before and two weeks after injection	4 visits	ADH, NDW, DSB
Mann et al. (2014) [27]	NTX 6 months	first fMRI was after withdrawal symptoms had subsided and the other 2 weeks after treatment beginning	1 year	ADS Score, OCDS Score, AUDIT, AUQ
Meyerhoff et al. (2018) [18]	GBP 1 week	MR spectroscopy after at least 1 week taking GBP	NA	SCID 2.0, LDH, CIWA
Morris et al. (2017) [31]	NTX 1 day	fMRI—2 h after NTX or placebo intake	NA	BDI-II, STAI
Myrick et al. (2008) [28]	NTX 8 days	fMRI on day 7 after at least 24 h of abstinence	NA	ADS Score, OCDS Score, TLFB, DD
Nestor et al. (2019) [36]	NTX 1 day	fMRI—2 h after NTX or placebo intake	The study has mentioned a follow-up, but not its length	ASSIST, TLFB
Priscindaro et al. (2021) [41]	GBP 16 weeks	MR spectroscopy were acquired before start of treatment and again approximately 14 days after randomization.	NA	CIWA
Savulich et al. (2017) [26]	NTX 4 weeks	4 fMRIs—2 h prior MRI NTX or PLA (4 days/times)	NA	WTAR, CTQ, PSS, AUDIT, BDI-II, STAI
Schacht et al. (2012) [14]	NTX 7 days	fMRI conducted on the sixth day of treatment	On the second visit	ADS Score, OCDS Score, DPD, HDD, DPDD, AASE, AI
Schacht et al. (2013) [40]	GBP + FMZ 6 weeks	fMRI was performed between the second and third week of treatment (mean scan day = 15; SD 2.5 days)	NA	ADS Score, OCDS Score, HDD, CIWA
Schacht et al. (2017) [13]	NTX 16 weeks	fMRI conducted at baseline and week 2	9 visits	ADS Score, OCDS Score, DPD, HDD, DPDD
Spagnolo et al. (2014) [30]	NTX 9 days	the fMRI was conducted on day 9	3 weeks	ADS Score, TLFB, ANDD, DD, HDD, DAPA
Umhau et al. (2010) [38]	ACA 4 weeks	H-MRS measures were obtained on the 4th and 25th day of the study	NA	ADS Score, TLFB, CIWA
Weerts et al. (2008) [29]	NTX 5 days	PET Scan before day 5 (no NTX) and on eighteenth day	Further evaluated in follow-up visits and continued naltrexone treatment	ADS Socre, ANDPDD, ANDDW

Alcohol Dependence Scale, ADS; Obsessive-Compulsive Drinking Scale, OCDS; Wechsler Test of Adult Reading, WTAR; Childhood Trauma Questionnaire, CTQ; Perceived Stress Scale, PSS; Alcohol Use Disorders Identification Test, AUDIT; The Beck Depression Inventory, BDI-II; Spielberger-State Anxiety Inventory, STAI; 9 item Patient Health Questionnaire, PHQ-9; Time Line Follow Back (for the past 7, 30 and 90 days), TLFB; Days Since Last Drink, DSLD; Pennsylvania Alcohol Craving Scale, PACS; Alcohol Urge Questionnaire, AUQ; Age Drink Heavily, ADH; Number of Drinks per Week, NDW; Days Sober at Baseline, DSB; Drinking Days (number), DD; Average Number Drinks per Day, ANDD; Heavy Drinking Days, HDD; Days Abstinent Prior Admission, DAPA; Drinks per Drinking Days, DPDD; Drinks per Day, DPD; Alcohol Intake (g/week), AI; Alcohol Abstinence Self-Efficacy, AASE; Multidimensional Alcohol Craving Scale, MACS; Time to First Heavy Drinking Day, TFHD; Average Number of Drinks per Drinking Day, ANDPDD; Average Number of Drinking Days per Week, ANDDPW; Lifetime Alcohol Consumption (in thousands), LTAC; Years of Heavy Drinking (number of years patients consumed an average of 560 g of ethanol weekly), YHD; Mean Alcohol Intake in the Preceding Year, MAIPY; Mean Time of Alcohol Abuse, MTAA; Clinical Institute Withdrawal Assessment, CIWA; Structured Clinical Interview for the DSM-IV 2.0, SCID 2.0; Lifetime Drinking History (LDH).

## Data Availability

All the supporting data are presented in the manuscript.

## Supplementary Materials

The following are available online at https://www.mdpi.com/article/10.3390/brainsci12030386/s1. Supplementary File 1: PRISMA statement checklist [54].
